# Physical exercise as a therapeutic approach for adults with insomnia: systematic review and meta-analysis

**DOI:** 10.31744/einstein_journal/2022AO8058

**Published:** 2022-07-18

**Authors:** Carolina Vicaria Rodrigues D’Aurea, Cristina Frange, Dalva Poyares, Altay Alves Lino de Souza, Mario Lenza

**Affiliations:** 1 Hospital Israelita Albert Einstein São Paulo SP Brazil Hospital Israelita Albert Einstein , São Paulo , SP , Brazil .; 2 Universidade Federal de São Paulo São Paulo SP Brazil Universidade Federal de São Paulo , São Paulo , SP , Brazil .

**Keywords:** Sleep, Sleep initiation and maintenance disorders, Yoga, Tai Ji, Exercise, Exercise movement techniques

## Abstract

**Objective:**

To systematically review the effects (benefits and harms) of different types of physical exercise on insomnia outcomes in adult populations with no mood disorders. Objective and subjective sleep outcomes and related mismatches were analyzed.

**Methods:**

Systematic review and meta-analysis. Quality of evidence was also examined.

**Results:**

Six studies including 295 participants with insomnia diagnosis were selected. Yoga, Tai Chi, resistance exercise and aerobic exercise were used in protocols with different duration, intensity and frequency. Studies involved different populations, including inactive or sedentary individuals, older adults and postmenopausal women. Physical exercise improved subjective sleep quality (very low quality of evidence) and reduced insomnia severity (high quality of evidence).

**Conclusion:**

Findings suggest individualized physical exercise must be addressed to design optimal protocols, with standardized type, duration, intensity, and frequency. For the time being, physical exercise may be considered an alternative and/or ancillary therapeutic modality for patients diagnosed with insomnia. Physical exercise can be used to improve subjective complaints, but not objective sleep outcomes.

## INTRODUCTION

Physical activity refers to any form of movement that results in energy expenditure and includes all activities of daily living, whether professional, domestic or recreational. Exercise is a structured form of physical activity defined by its intensity, duration and frequency. ^( 1 )^

Physical activity and exercise have been attracting attention as non-pharmacological treatment alternatives for patients with insomnia, ^( 2 )^ since physically active people have better sleep quality, less sleep-related complaints and less daytime sleepiness and fatigue. ^( 3 )^ In healthy adults, regular exercise improves subjective ^( 4 - 6 )^ and objective sleep quality parameters such as total sleep time (TST), sleep efficiency (SE), slow wave sleep (SWS) and sleep onset latency (SOL), ^( 7 )^ which can be measured using polysomnography (PSG) or actigraphy (ACT).

In spite of well-established positive effects of physical activity and exercise on sleep, a previous meta-analysis examining the impacts of physical activity on insomnia outcomes revealed that acute bouts of exercise had small beneficial effects on TST, SOL, SE, non–rapid eye movement (NREM) sleep stages N1 and N3 (or SWS), and rapid eye movement (REM) sleep, or R stage. Moderate beneficial effects on wake after sleep onset (WASO) have also been reported. In contrast, regular exercise had small beneficial effects on TST and SE, small to moderate beneficial effects on SOL and moderate beneficial effects on sleep quality. ^( 5 )^

Many researchers have investigated the effects of physical activity and exercise on insomnia. In spite of low quality of evidence, the European Guideline for the Diagnosis and Treatment of Insomnia suggests that exercise can be an effective therapeutic strategy in patients with insomnia. Therefore, further investigations are warranted. ^( 8 )^ According to a more recent meta-analysis, exercise improves subjective sleep quality ^( 6 )^ in patients with insomnia.

Insomnia is defined as subjective perception of poor night time sleep, including difficulty initiating or maintaining sleep, early morning wakening at least three times per week over a period of three months and daytime impairments such as a lack of energy, attention or memory difficulties, cognitive dysfunction, fatigue and somatic symptoms. ^( 9 )^ Cognitive behavioral therapy for insomnia (CBT-I) is the recommended first-line treatment for the condition. ^( 8 )^ Several medications can also be used. However, side effects are common. ^( 10 )^ The diagnosis of insomnia relies on medical history ( *i.e* ., subjective data). Hence, sleep patterns (sleep-wake cycle) and perception of sleep must be taken into account in insomnia diagnosis and outcome assessment. Subjective parameters are of primary importance in the diagnosis of insomnia and PSG is not routinely recommended, except to rule out other sleep disorders.

The perception of sleep varies widely among insomnia patients. These patients often underestimate TST and overestimate SOL and nocturnal awakenings WASO when compared to objective sleep findings. ^( 11 - 14 )^ This discrepancy in subjective perception of insomnia symptoms is a common feature in insomnia patients. ^( 11 , 14 )^

A major advantage of using exercise as a nonpharmacological treatment for insomnia is the positive side effect on health and well-being. Regular exercise has been shown to facilitate weight loss, prevent pain and improve mood and sleep quality in patients with insomnia. Also, potential positive impacts on exercise performance create a positive circle. ^( 15 - 18 )^ In contrast, lack of regular physical activity may contribute to a wide range of conditions, including insomnia. The consensus is that sufficient sleep and adequate physical activity levels are vital to maintain good quality of life. This recommendation is supported by the World Health Organization (WHO). ^( 19 )^

The effects of exercise on insomnia have been investigated in prior studies in which mood disorders were a confounding factor. ^( 6 , 20 , 21 )^ Physical exercise interventions were considered effective whenever the following goals were met: improvement in sleep quality and/or duration and improvement of insomnia symptoms (SOL <30 minutes and/or WASO <30 minutes and/or decreased frequency of awakenings or other sleep complaints and/or TST >6 hours and/or SE >85%). ^( 22 )^ To the best of our knowledge, systematic reviews aimed specifically at examining the effects of exercise on insomnia patients who do not suffer from mood disorders have not been carried out to date.

## OBJECTIVE

To systematically review the effects (benefits and harms) of different types of physical exercise on insomnia outcomes in adult populations with no mood disorders.

## METHODS

This systematic review was registered with the Project Management System (SGPP - *Sistema Gerenciador de Projeto de Pesquis* a) of *Hospital Israelita Albert Einstein* (HIAE) (# 2666-16) and the Prospective International Registry of Systematic Reviews (PROSPERO, # CRD42017053274). Preferred Reporting Items for Systematic Reviews and Meta-Analyses (PRISMA) ^( 23 )^ and the Cochrane Handbook for Systematic Reviews of Interventions ^( 24 )^ guidelines were followed.

### Literature search

Five electronic databases (PubMed, EMBASE, Cochrane Library, Scopus and LILACS) were searched for randomized controlled trials (RCTs) and quasi-RCTs. There were no restrictions regarding date of article publication (from inception date to May, 2021) or language in this review and meta-analysis. The general search strategy was based on controlled vocabulary (Medical Subject Headings in PubMed) and the following key words: (“exercise” OR “physical activity” OR “exercise movement techniques” OR “Tai Chi” OR “muscle stretching exercises” OR “aerobic exercise” OR “resistance training” OR “yoga” OR “pilates”) AND (“sleep initiation and maintenance disorders” OR “sleep wake disorders” OR “wakefulness” OR “insomnia” OR “sleep initiation difficulties” OR “sleep initiation insomnia”). No filters were used. The WHO International Clinical Trials Records Platform and registrations within *ClinicalTrials.gov* were also searched for identification of ongoing or recently completed studies.

### Inclusion and exclusion criteria

Inclusion criteria were as follows:

Types of studies: RCTs and quasi-RCTs in which participants had been randomly allocated to an Intervention or a Control Group.

Type of participants: adults (≥18 years of age) of both sexes diagnosed with insomnia according to standard diagnostic criteria such as the Diagnostic and Statistical Manual of Mental Disorders (DSM-V) and the International Classification of Sleep Disorders (ICSD) or the Research Diagnostic Criteria ^( 25 )^ for insomnia, to standard measures of sleep and insomnia such as the Pittsburgh Sleep Quality Index (PSQI), ^( 26 , 27 )^ Insomnia Severity Index (ISI) ^( 28 )^ and the Athens Insomnia Scale (AIS), ^( 7 )^ or to sleep logs/diary or ACT data. Insomnia symptoms were not accounted for in this review.

Types of interventions: different types of physical exercise protocols ( *e.g* ., stretching exercises, aerobic exercises, resistance exercises and mind-based techniques such as Tai Chi and Yoga, which consist primarily of isometric exercises) used to treat insomnia.

Types of outcome measures: TST, SOL, SE and WASO measured using PSG or ACT, or sleep PSQI and insomnia AIS, ISI scales. Sleep logs/diaries analyzing TST, SOL, SE and WASO data were included as a secondary outcome measure.

Studies including participants with depressive symptoms, psychiatric or other medical disorders, or other sleep disorders, such as sleep apnea and restless legs syndrome, were excluded.

### Data extraction

Titles and abstracts were examined independently by two reviewers. then compared using data extraction sheets. Disagreements regarding study eligibility were settled by a third author. Full-text articles were read whenever abstracts were unclear. Following full-text screening, references listed in identified texts were searched manually for identification of additional manuscripts. Reviewers were not blinded to author names, institutions or journal of publication. Authors were contacted for additional data.

### Methodological quality assessment

The quality of studies included in the meta-analysis was assessed using the Cochrane Handbook for Systematic Reviews of Interventions ^( 24 )^ criteria. The risk of bias was assessed independently by two researchers. Disagreements were settled by a third author. Selected studies were assessed for methodological quality regarding adequacy of randomization, allocation concealment, blinding of participants and outcome assessors, incomplete outcome data, selective publication and other potential risks of bias ( *e.g* ., unbalanced demographic data, inappropriate funding and conflicts of interest).

The Grading of Recommendations Assessment, Development and Evaluation (GRADE) ^( 29 )^ classification method was used to assess the quality of evidence and the strength of recommendations associated with primary outcomes.

### Statistical analysis

Data were pooled using a random-effects model constructed using Review Manager ^®^ software, version 5.3 (The Nordic Cochrane Center, The Cochrane Collaboration, Copenhagen, Denmark, 2012) and accounting for potential heterogeneity in RCTs. A confidence interval of 95% (95%CI) was adopted. For continuous data (PSQI, ISI, TST, SE, SOL, WASO) the standardized mean difference (SMD) or the mean difference (MD) and the respective 95%CI were calculated, *as per* Cochrane handbook guidelines. ^( 30 )^ The MD was used whenever data included in the meta-analysis were derived from the same indicator. The SMD was used when data were derived from different indicators. Heterogeneity was assessed by visual inspection of graphs (forest plots) and statistical tests for heterogeneity (I ^2^ ). Heterogeneity was interpreted according to the rough guide recommended in the Cochrane handbook: ^( 31 )^ 0-40% might not be important; 30-60% may represent moderate heterogeneity; 50-90% may represent substantial heterogeneity and 75-100% considerable heterogeneity. Whenever heterogeneity was detected (I ^2^ >50%), efforts were made to identify potential causes. In significant cases, individual characteristics of studies were compared and sensitivity analysis performed. Whenever necessary, the standard deviation ^( 32 )^ was calculated from: SE; 95%CI and sample size from the p value and the number of individuals in each group. ^( 31 )^ For the meta-analysis, participants in selected studies were pooled and compared according to the following exercise categories: control *versus* aerobic exercise, resistance exercise, Yoga or Tai Chi.

## RESULTS

### Study selection

**Figure 1:**
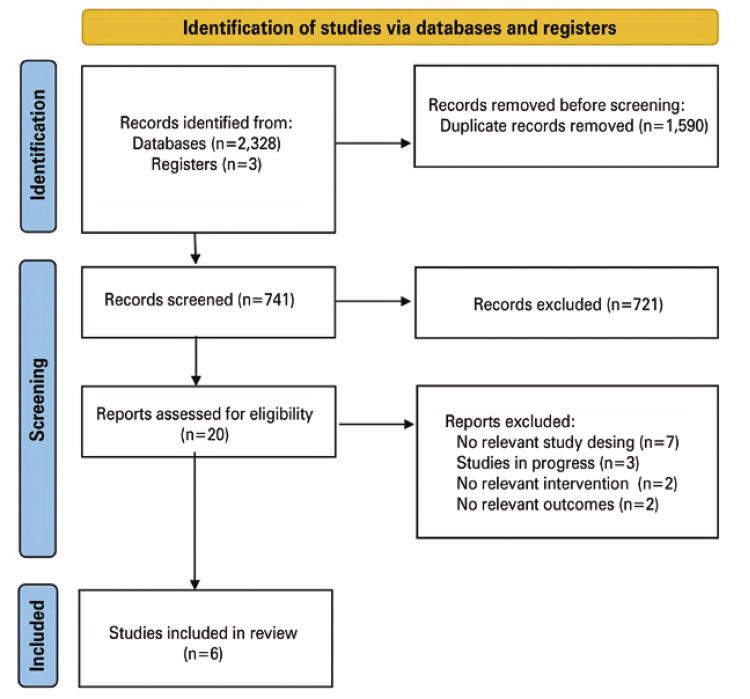
Flow chart (PRISMA) depicting results of the systematic literature search following application of inclusion and exclusion criteria

The initial database search yielded a total of 2,331 records. After removal of duplicates, 741 articles remained. Of these, 721 failed to meet inclusion criteria following title and abstract analysis and were excluded. Twenty articles were analyzed in full, six of which met selection criteria (295 participants in total) ( Figure 1 ) and were included in the review and the meta-analysis.

### Characteristics of selected studies

The characteristics of studies included in the review are shown in table 1 . Overall, the systematic review comprised 295 individuals from three countries (Brazil, ^( 15 , 33 , 34 )^ United Kingdom ^( 35 )^ and United States of America), ^( 36 , 37 )^ who were aged 30 to 85 years ( Table 1 ). In five studies, participant recruitment was based on a clinical diagnosis of insomnia ^( 15 , 33 , 34 , 36 , 37 )^ obtained via a structured interview conducted by a sleep medicine specialist, *as per* Diagnostic and Statistical Manual of Mental Disorders (DSM-IV) ^( 38 )^ and/or International Classification of Sleep Disorders (ICSD-2) criteria. In the remaining study, enrollment was based on research diagnostic criteria and whether this tool was applied by a physician was not reported. ^( 35 )^

**Table 1:** Summary of articles included in the review and the meta-analysis

Studies	n	Mean age (±SD) or range	Insomnia classification	Intervention(s) and controls/duration	Outcomes
Passos et al. ^(15)^ Brazil	48	Inactive adults Both sexes 44.4 (±8.0)	Clinical diagnosis of insomnia (DSM-IV + ICSD-2)	4 groups: G1 = moderate-intensity aerobic exercise (50 minutes, 1 session/week) G2 = high-intensity aerobic exercise (50 minutes, 1 session/week) G3 = moderate-intensity resistance exercise (50 minutes, 1 session/week) Ctrl = no treatment	PSG: TST, SE, SOL, WASO Sleep diary: TST, SE, SOL, WASO
Reid et al. ^(36)^ United States of America	17	Sedentary adults Both sexes 61.6 (± 4.3)	Clinical diagnosis of insomnia (ACT + 7 day sleep diary)	2 groups: G1 = moderate aerobic physical activity+ sleep hygiene (increasing from 10 to 40 minutes each week, 4 sessions/week) Ctrl = sleep hygiene 4 months	PSQI Epworth Sleepiness Scale SF-36
Afonso et al. ^(33)^ Brazil	44	Postmenopausal women 50-65	Clinical diagnosis of insomnia	3 groups: G1 = yoga (1 hour, 2 sessions/week) G2 = passive stretching (1 hour, 1 session/week) Ctrl = no treatment 4 months	PSG: TST, SE, SOL, WASO ISI MENQOL
Irwin et al. ^(37)^ United States of America	123	Older adults Both sexes 55-85	Clinical diagnosis of insomnia (DSM-IV + ICSD-2)	3 groups: G1 = CBT (2 hours, 2 sessions/week) G2 =Tai Chi (2 hours, 2 sessions/week) Ctrl = sleep seminar education + sleep hygiene (2 hours, 2 sessions/week) 4 months	PSG: TST, SE, SOL, WASO Sleep diary: TST, SE, SOL, WASO PSQI Epworth Sleepiness Scale AIS
Hartescu et al. ^(35)^ United Kingdom	41	Inactive adults Both sexes 59.80 (±9.5)	Clinical diagnosis of insomnia (Research Diagnostic Criteria)	2 groups: G1 = moderate to vigorous physical activity (≥150 minutes, 1 session/week) Ctrl = no treatment 6 months	Epworth Sleepiness Scale ISI EQ5D-5L
D’Aurea et al. ^(34)^ Brazil	28	Inactive adults Both sexes 30-55	Clinical diagnosis of insomnia	3 groups: G1 = resistance exercise (1 hour, 3 sessions/week) G2 = low-intensity stretching (1 hour, 3 sessions/week) Ctrl = no treatment 4 months	PSG: TST, SE, SOL, WASO ACT: TST, SE, SOL, WASO PSQI ISI SF-36

SD: standard deviation; DSM-IV: Diagnostic and Statistical Manual of Mental Disorders; ICSD-2: International Classification of Sleep Disorders; G: group; Ctrl: Control Group; PSG: polysomnography; TST: total sleep time; SE: sleep efficiency; SOL: sleep onset latency; WASO: wake after sleep onset; ACT: actigraphy; PSQI: Pittsburgh Sleep Quality Index; SF-36: Short Form Health Survey 36; ISI: Insomnia Severity Index; MENQOL: Menopause-specific Quality of Life Questionnaire; CBT: cognitive behavioral therapy; AIS: Athens Insomnia Scale; EQ5D-5L:EUROQOL5D5L.

Exercise interventions ranged from mind-based exercise ( *e.g* ., Yoga and Tai Chi) to aerobic and resistance exercises, with different levels of intensity, duration and frequency. In studies involving moderate intensity aerobic exercise, walking ^( 35 )^ or a treadmill was used. ^( 36 )^

### Bias assessment

**Figure 2:**
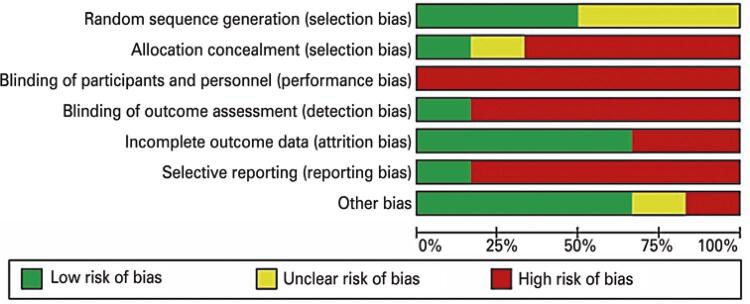
Classification of risk of bias expressed as percentages across selected studies

The risk of bias in the different studies was high. Domains with higher risk of bias were blinding of participants and researchers, blinding of outcome assessment, allocation concealment and incomplete outcome data ( Figure 2 ).

Generation of sequence of randomization: three studies failed to describe methods used for random sequence generation ^( 15 , 33 , 36 )^ and therefore had unclear risk of bias. The three remaining studies used stratified, block ^( 35 , 37 )^ or computer randomization and were classified as low risk of bias. ^( 34 )^

Allocation concealment: four studies were carried out without allocation concealment and therefore had a high risk of bias. ^( 15 , 33 , 34 , 36 )^ One study had unclear risk of bias, since not enough details were provided so as to ensure concealment was used. ^( 35 )^ Only one study reported that members of the research team who evaluated participants had access to the randomization list. That study fell into the low risk of bias category. ^( 37 )^

Blinding of participants and personnel/researchers: articles selected had a high risk of bias, as participants and research team members were not blinded.

Blinding of outcome assessment: articles had a high risk of bias, with the exception of one, in which outcome assessors were unaware of group attributions. ^( 37 )^

Incomplete outcome data: studies fell into the low risk of bias category whenever the following conditions were met: more than 80% of participants completed follow-up, drop outs were explained and balanced between groups and intention-to-treat analysis was reported. Two studies had a high risk of attrition bias, as less than 80% of participants completed follow-up. ^( 33 , 36 )^ In the four remaining studies, drop outs were explained and balanced and cases submitted to appropriate statistical analysis. ^( 15 , 34 , 35 , 37 )^ Therefore, these were classified as low risk of bias.

Selective reporting: one study was classified as low risk of bias, because it included all primary outcomes of interest prespecified in this review. ^( 34 )^ Remaining studies were classified as high risk of bias, since results associated with outcomes of interest set for this review were not presented or partially presented. ^( 15 , 33 , 35 - 37 )^

Other biases: four studies had low risk of bias ^( 15 , 34 , 36 , 37 )^ and two studies had unclear risk of bias (one due to insufficient information to assess bias with regard to these factors ^( 33 )^ and the other due to lack of baseline outcome data to determine whether groups were balanced prior to the intervention). ^( 35 )^ Risk of bias associated with the influence of sponsors was not detected.

### Effects of interventions

The effects of type of physical exercise (aerobic exercise, yoga, resistance exercise, stretching exercise and Tai Chi) relative to the Control Group are shown in table 2 .

**Table 2:** Summary of findings for the main comparison

Effects of physical exercise in insomnia and control patients					
Patient or population: adults with insomnia Intervention: physical exercise Comparison: Control Group (no activity)					
Outcomes	Illustrative comparative risks* (95%CI)		Number of participants (studies)	Quality of evidence (GRADE)	Comments
	Assumed risk	Corresponding risk			
	Control Group	Physical Exercise			
PSG - TST; PSG is the gold standard for analysis of physiological variables affecting sleep. Follow-up: 4 months	Mean TST in Control Groups was 341.3 minutes	SMD 0.09 lower (0.45 lower to 0.28 higher)	121 participants (3 studies)	⊕⊕⊝⊝ ^1,2,3^ Low	Primary outcome, difference was not statistically significant
PSG - SOL Follow-up: 4 months	Mean SOL in Control Groups was 21.06 minutes	MD 0.8 higher (6.13 lower to 7.73 higher)	145 participants (4 studies)	⊕⊝⊝⊝ ^2,3,4^ Very low	Primary outcome, difference was not statistically significant
PSG - SE Follow-up: 4 months	Mean SE in Control Groups was 79.02%	MD 0.9 lower (4.52 lower to 2.73 higher)	145 participants (4 studies)	⊕⊕⊝⊝ ^2,4^ Low	Primary outcome, difference was not statistically significant
PSG - WASO Follow-up: 4 months	Mean WASO in Control Groups was 66.03 minutes	MD 0.79 higher (13.95 lower to 15.52 higher)	145 participants (4 studies)	⊕⊝⊝⊝ ^2,3,4^ Very low	Primary outcome, difference was not statistically significant
Sleep questionnaire – Insomnia Insomnia Severity Index (0 to 28 points, 0 is best). Follow-up: 4 months	Mean score in sleep questionnaire - insomnia in Control Groups was 9.36	SMD 0.72 lower (1.1 lower to 0.34 lower)	121 participants (3 studies)	⊕⊕⊕⊕ ^1,2^ High	Primary outcome, difference was statistically significant
Sleep questionnaire - Pittsburgh Sleep Questionnaire Index (0 to 21 points, >5 indicates good sleep quality). Follow-up: 4 months	Mean score in sleep questionnaire - PSQI in Control Groups was 6.83	MD 3.17 lower (4.23 lower to 2.12 lower)	108 participants (3 studies)	⊕⊝⊝⊝ ^1,2,3,5^ Very low	Primary outcome, difference was statistically significant
Sleep diary - TST Follow-up: 4 months	Mean TST according to sleep diary in Control Groups was 334.25 minutes	MD 47.72 higher (46.92 higher to 48.52 higher)	97 participants (2 studies)	⊕⊕⊕⊝ ^1,2,5^ Moderate	Secondary outcome, difference was statistically significant

* The basis for the assumed risk ( *e.g* . the median Control Group risk across studies) is provided in footnotes. The corresponding risk (and its 95%CI) is based on the assumed risk in the comparison group and the relative effect of the intervention (and its 95%CI).

GRADE Working Group grades of evidence.

High quality: further research is very unlikely to change our confidence in the estimate of effect.

Moderate quality: further research is likely to have an important impact on our confidence in the estimate of effect and may change the estimate.

Low quality: further research is very likely to have an important impact on our confidence in the estimate of effect and is likely to change the estimate.

Very low quality: we are very uncertain about the estimate.

1. Risk of bias: all studies had some methodological flaws and results involved a risk of bias.

2. Indirectness: the total number of events and/or number of participants was small; although outcomes are similar, interventions differ among studies.

3. Imprecision: very large confidence interval, with high level of imprecision.

4. Risk of bias: all studies had serious methodological flaws and results involved a high risk of bias.

5. Inconsistency: there was considerable heterogeneity.

95%CI: confidence interval 95%; RR: risk ratio; TST: total sleep time; SE: sleep efficiency; SOL: sleep onset latency; PSG: polysomnography; WASO: wake after sleep onset; SMD: standardized mean difference; MD: mean difference; PSQI: Pittsburgh Sleep Quality Index.

**Figure 3:**
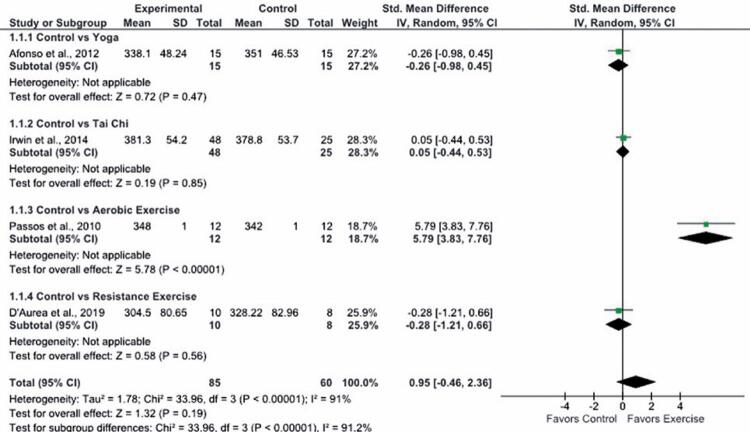
Forest plot illustrating 95% confidence intervals derived from studies examining the effect of exercise on total sleep time measured using polysomnography (n=145)

Total sleep time before and after the intervention varied between studies. Comparisons between Intervention and Control Groups revealed the following TST averages: Yoga *versus* control, 338.1 and 351 minutes respectively; ^( 33 )^ resistance exercise *versus* control, 304.5 and 328.2 minutes respectively; ^( 34 )^ Tai Chi *versus* control, 381.3 and 378.8 minutes respectively; ^( 37 )^ aerobic exercise *versus* control, 348 and 342 minutes respectively. ^( 15 )^ Aerobic exercise was the only intervention associated with a statistically significant increase in TST in the Exercise Group after the intervention. Interventions were not associated with statistically significant TST improvement (SMD, 0.95 minutes; 95%CI: -0.46-2.36). High statistical heterogeneity was detected (Tau ^2^ =1.78, I ^2^ =91%) ( Figure 3 ). Therefore, subgroup analysis was performed. In this analysis, one study ^( 15 )^ was excluded, since it involved an acute intervention (a single session of physical exercise), whereas in remaining studies interventions lasted four months. Subgroup analysis failed to show statistically significant differences in TST between physical exercise and Control Groups (SMD, -0.09 minutes; 95%CI: -0.45-0.28).

SD: standard deviation; 95%CI: 95% confidence interval.

Sleep onset latency was investigated in four studies comparing exercise and control. Findings were as follows: Yoga and control, 26.45 and 14.95 minutes respectively; ^( 33 )^ resistance exercise and control, 21.99 and 27.86 minutes respectively; ^( 34 )^ Tai Chi and control, 19 and 22.3 minutes respectively; ^( 37 )^ acute aerobic exercise and control, 16.8 and 13.8 minutes respectively. ^( 15 )^ Acute aerobic exercise ^( 15 )^ was the only intervention associated with a statistically significant increase in SOL. In remaining studies, interventions failed to promote statistically significant improvements in SOL (MD, 0.8 minutes; 95%CI: -6.13, -7.73). No statistical heterogeneity was indicated (χ ^2^ =2.43; I ^2^ =0%).

Sleep efficiency increased in Exercise Groups compared to controls (differences were as follows: Yoga and control, 78.10% and 82.31% respectively; ^( 33 )^ resistance exercise and control 76.51% and 75.43% respectively; ^( 34 )^ Tai Chi and control, 80.6% and 79.5%. ^( 37 )^ With the exception of acute aerobic exercise (80.9% relative to 81.9% in the Control Group), ^( 15 )^ which was associated with statistically significant differences, interventions were not associated with statistically significant improvements in sleep efficiency (MD, -0.90%; 95%CI: -4.52-2.73). No heterogeneity was detected (χ ^2^ =1.67; I ^2^ =0%).

With regard to WASO, studies revealed mixed results after comparative analysis of Exercise and Control Groups, as follows: Yoga and control, 69.51 and 62.12 minutes respectively; ^( 33 )^ resistance exercise and control, 56.83 and 71.38 minutes respectively; ^( 34 )^ Tai Chi and control, 72.5 and 75.1 minutes respectively; ^( 37 )^ acute aerobic exercise and control 65.3 and 60.7 minutes respectively. ^( 15 )^ Acute aerobic exercise led to a statistically significant decrease in WASO. No statistically significant improvements were obtained following interventions (MD, 0.79 minutes; 95%CI: -13.95-15.52) and no heterogeneity was detected (χ ^2^ =0.72; I ^2^ =0%).

With regard to subjective sleep quality assessment, the PSQI was used in three studies comparing exercise and Control Groups. Findings were as follows: resistance exercise and control, 7.4 and 12.6 ^( 34 )^ (poor sleep quality and presence of sleep disturbances respectively); Tai Chi and control, 8 and 9.6 respectively ^( 37 )^ (poor sleep quality in both cases); aerobic exercise and control, 5.1 and 9.5 ^( 36 )^ (good/poor and poor sleep quality respectively). Interventions had a significant impact on sleep quality (MD, -3.17 points lower in Intervention Groups; 95%CI: -4.23, -2.12 points lower). Lower PSQI scores indicate better sleep quality. Substantial heterogeneity was observed (χ ^2^ =8.17; I ^2^ =76%). To address this heterogeneity, only the study carried out by Irwin et al. ^( 37 )^ was considered, since sample size was larger in that relative to remaining studies and methodology was better, with a lower risk of bias. This led to a statistically significant improvement in sleep quality (MD, -1.60 points; 95%CI: -3.12, -0.08).

**Figure 4:**
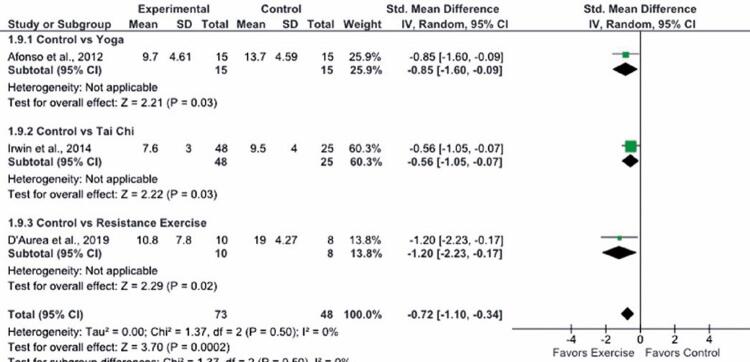
Forest plot illustrating 95% confidence intervals derived from three studies examining the effects of exercise on subjective perception of insomnia *as per* the Insomnia Severity Index and the Athens Insomnia Scale (n=121)

The ISI and the ASI were used to assess subjective insomnia in two and one study respectively. Scores were as follows: Yoga *versus* control ISI, 9.7 and 13.7 respectively; ^( 33 )^ resistance exercise *versus* control ISI, 10.8 and 19 respectively; ^( 34 )^ Tai Chi *versus* control ASI, 7.6 and 9.5 respectively. ^( 37 )^ Scores decreased significantly in all cases, indicating improved subjective perception of insomnia. The meta-analysis revealed a statistically significant decrease in insomnia symptoms following interventions. However, improvements were not clinically significant (SMD, -0.72 points; 95%CI: -1.10, -0.34). No statistical heterogeneity was detected (χ ^2^ =1.37; I ^2^ =0%) ( Figure 4 ).

SD: standard deviation; 95%CI: 95% confidence interval.

### Quality of evidence

The level of certainty of the evidence ranged from very low to moderate. High level of certainty was limited to the ISI outcome ( Table 3 ).

**Table 3:** Grading of Recommendations Assessment, Development and Evaluations (GRADE) summary of outcomes of selected randomized controlled trials

Outcomes	Number of studies	Risk of bias	Indirect evidence	Inaccuracy	Inconsistency	Number of participants	Relative effect (95%CI)	Quality of evidence (GRADE)	Clinical outcome, significance
TST minutes (PSG)	3	Severe ^1^	Severe ^2^	Severe ^3^	Not severe	121	SMD: 0.95 (-0.46-2.36)	^⨁◯◯◯^ Very low	Primary outcome, difference was not statistically significant
SOL minutes (PSG)	4	Very severe ^4^	Severe ^2^	Severe ^3^	Not severe	145	MD: 0.8 (-6.13-7.73)	^⨁⨁◯◯^ Low	Primary outcome, difference was not statistically significant
SE % (PSG)	4	Very severe ^4^	Severe ^2^	Not severe	Not severe	145	MD: -0.9 (-4.52-2.73)	^⨁◯◯◯^ Very low	Primary outcome, difference was not statistically significant
WASO minutes (PSG)	4	Very severe ^4^	Severe ^2^	severe ^3^	Not severe	145	MD: - 0.79 (-13.95-15.52)	^⨁⨁◯◯^ Low	Primary outcome, difference was not statistically significant
Severity of insomnia	3	Severe ^1^	Severe ^2^	Not severe	Not severe	121	SMD: -0.72 (-1.1, -0.34)	^⨁⨁⨁⨁^ High	Primary outcome, difference was statistically significant
Sleep quality (PSQI)	3	Severe ^1^	Severe ^2^	Severe ^3^	Very severe ^5^	108	MD: -3.17 (-4.23, -2.12)	^⨁◯◯◯^ Very low	Primary outcome, difference was statistically significant

GRADE Working Group grades of evidence.

High quality: further research is very unlikely to change our confidence in the estimate of effect.

Moderate quality: further research is likely to have an important impact on our confidence in the estimate of effect and may change the estimate.

Low quality: further research is very likely to have an important impact on our confidence in the estimate of effect and is likely to change the estimate.

Very low quality: we are very uncertain about the estimate.

1. Risk of bias: all studies had some methodological flaws and results involved a risk of bias.

2. Indirectness: the total number of events and/or number of participants was small; although outcomes are similar, interventions differ among studies.

3. Imprecision: very large confidence interval, with high level of imprecision.

4. Risk of bias: all studies had serious methodological flaws and results involved a high risk of bias.

5. Inconsistency: there was considerable heterogeneity.

PSG: polysomnography; PSQI: Pittsburgh Sleep Quality Index; MD: mean difference; SMD: standardized mean difference; TST: total sleep time; SE: sleep efficiency; SOL: sleep onset latency; WASO: wake after sleep onset.

The quality of evidence regarding the inability of physical exercise to improve sleep quality relative to the Control Group after a 4-month intervention period was very low. Poor quality of evidence was due to risks of severe bias, indirect evidence and inaccuracy, and very severe inconsistency.

Low quality of evidence also indicated that, compared to the Control Group, physical exercise did not improve SOL measured by PSG after a 4-month intervention period. The quality of evidence was downgraded by the risk of very severe bias and severe indirect evidence and inaccuracy.

Low quality of evidence suggested that, compared to the Control Group, physical exercise did not improve WASO measured by PSG after a 4-month intervention period. The quality of evidence was downgraded by the risk of very severe bias and severe indirect evidence and inaccuracy. High quality evidence indicated physical exercise significantly reduced insomnia severity ^( 39 )^ after a 4-month intervention period.

## DISCUSSION

This systematic review set out to investigate the effects of exercise on insomnia, sleep patterns and subjective perception of sleep in participants. Results showed that physical exercise had a positive effect on subjective sleep quality and insomnia severity, but no impact on objective sleep parameters measured by PSG or ACT.

Overall, studies were of low quality, involved several risks of bias and had small sample size. When compared to the Control Group, physical exercise had a positive impact on subjective sleep measures. However, evidence (mostly low quality) suggests physical exercise does not improve objective sleep parameters measured by PSG (TST, SOL and WASO) or sleep quality measured by questionnaires. Low quality of evidence was due to severe bias, indirect evidence and inaccuracy.

In this study, exercise was associated with statistically significant improvements in subjective insomnia parameters, but not in objective sleep parameters. This subjective-objective mismatch is a common characteristic of insomnia reported in literature. ^( 11 , 39 - 43 )^ Nonetheless, improvement in subjective sleep perception is one of the main clinical outcomes of insomnia treatment. ^( 22 )^ In patients with appropriately and objectively measured sleep parameters, disproportionally severe subjective complaint of insomnia has attracted the attention of researchers and was named “misperception of sleep”, ^( 37 )^ “subjective insomnia” or “paradoxical insomnia”. ^( 44 - 46 )^

A study carried out by a group of researchers in Pennsylvania reported two main phenotypes of insomnia patients: with or without objectively short TST. ^( 47 )^ Given the different insomnia phenotypes, apparent discrepancies between subjective and objective improvements might be expected in insomnia patients with different phenotypes. Moreover, insomnia is thought to be a disorder of hyperarousal, which may interfere with the ability to initiate and/or maintain sleep. Insomnia has also been shown to play a role in sleep initiation and perception. ^( 47 - 49 )^

Recognition of subjective-objective mismatch has important implications from a diagnostic and therapeutic perspective. Lack of significant changes in objective sleep parameters should not be considered a primary outcome in insomnia patients. Assessing the sleep experience of a patient in laboratory settings may provide useful information for patients and treatment providers by showing to what extent the mismatch might be relevant to that individual.

Findings of this study are in keeping with recent literature demonstrating inconsistent subjective and objective results regarding the effects of exercise on sleep. ^( 6 , 50 , 51 )^ Despite the lack of differences in “objectively probable insomnia”, statistically significant differences detected in subjective sleep parameters associated with insomnia severity and sleep quality were in favor of exercise. Differences in insomnia severity suggest a clinically significant improvement, since pooled data indicated a moderate positive effect of exercise, with an increase in ISI scores greater than 7 points, which is thought to be clinically significant. ^( 52 )^ As to sleep quality, a three-point change in PSQI scores is considered indicative of a minimum clinically significant difference. ^( 53 )^ In this study, PSQI scores dropped by 3.17 points. Therefore, the positive effect of exercise on sleep quality (very low quality of evidence) was thought to be small. No clinically significant improvements in sleep quality were observed following correction for heterogeneity. This may have been due to differences in exercise type, intensity and duration, pre-conditioning of participants prior to interventions, time of the day at which exercise was performed and warm up/cool down at the beginning and end of exercise sessions, which may have influenced relaxation and thus interfered with insomnia outcomes.

Results of this review and meta-analysis are significant, as they suggest exercise was an effective treatment for sleep quality and decrease insomnia severity in individuals with insomnia.

A significant amount of research has been undertaken to investigate the complex interrelationship between sleep and exercise. Many regulatory mechanisms have been proposed to explain the influence of exercise on sleep, and support the use of exercise as a treatment approach (or ancillary treatment approach) for insomnia. Regular exercise, particularly in the morning and afternoon, may elevate body temperature by a few degrees. When body temperature falls back to normal, drowsiness may be induced, promoting sleep. ^( 54 - 57 )^ Regular exercise may also increase melatonin secretion and improve sleep quality. ^( 58 )^ Exercise may reduce inflammation, boost the immune function ^( 59 )^ and enhance metabolic and endocrine functions by affecting growth hormone and cortisol levels, thereby inducing increased glucose utilization during REM sleep and increasing glucose levels in the evening, with reduced insulin sensitivity. ^( 60 )^ Exercise may affect autonomic function/heart rate variability ^( 54 )^ by mechanisms such as improved vagal modulation, leading to a decrease in heart rate, ^( 61 )^ with potentially beneficial effects, since autonomic hyperarousal plays a major role in the pathogenesis of insomnia. Exercise may affect important neurotransmitters involved in sleep regulation. ^( 5 , 54 )^ Exercise may have an “anti-depressant effect” and improve mood states, ^( 62 , 63 )^ promoting elevated levels of brain-derived neurotrophic factor (BDNF) and improving sleep quality. ^( 4 , 64 )^ Exercise may exert sleep pressure on the homeostatic dysfunction associated with insomnia. ^( 65 )^ These mechanisms have positive effects on health and possibly on sleep disorders such as insomnia. Also, exercise may be a promising ancillary therapeutic modality to CBT-I and therefore warrants further investigation.

This study has several strengths, including careful and rigorous screening, extraction and scoring processes and extensive subgroup analyses to explore the heterogeneity of results. In addition, all RCTs included reported insomnia diagnosis rather than insomnia symptoms. The study also has some limitations, which must be discussed. Firstly, the variety of exercise types (aerobic, resistance and mind-based) may have introduced a bias. Some of studies selected did not provide details about exercise interventions, such as type of aerobic exercise ( *e.g* ., walking, cycling, swimming), whether a warm up and cool down was included and exercise duration. However, since there is no standard exercise protocol, these differences may be inherent to studies of this kind. Secondly, the inclusion of exercise protocols with different duration may have impacted sleep in different ways. Another potential limitation is the inclusion of mind-based physical exercise as well as aerobic and resistance exercise modalities in the review. These may have had different impacts on sleep, since some also included relaxation techniques. Further studies with longer follow-up (>4 months) are warranted, preferably including subgroups analysis and accounting for patient age and exercise type and intensity, in order to identify subpopulations of insomnia patients that may benefit from the practice of exercise.

## CONCLUSION

In conclusion, in this review and meta-analysis, exercise improved subjective sleep quality (very low quality of evidence) and reduced insomnia severity (high quality of evidence). The value of the Insomnia Severity Index and the Athens Insomnia Scale in the assessment of insomnia was emphasized. However, physical activity did not lead to significant improvements in objective sleep parameters measured using polysomnography or actigraphy. Overall, the evidence was of very low quality. The conclusion was that individualized physical activity may be a safe and effective therapeutic alternative and/or ancillary treatment for some insomnia patients.
